# The management of exercise-induced anaphylaxis in a Chinese child with biologics: a case report

**DOI:** 10.3389/falgy.2024.1453873

**Published:** 2024-09-19

**Authors:** Nannan Jiang, Li Xiang, Huijie Huang, Xudong Zhang

**Affiliations:** ^1^ Department of Allergy, Beijing Children’s Hospital, National Center for Children's Health, Capital Medical University, Beijing, China; ^2^ Key Laboratory of Major Diseases in Children, Ministry of Education, Beijing, China

**Keywords:** omalizumab, exercise-induced anaphylaxis, lipid transfer protein, children, dupilumab

## Abstract

Exercise-induced anaphylaxis (EIA) is a rare and potentially life-threatening disorder. In difficult to control and refractory cases of EIA, biologics such as omalizumab and dupilumab have shown promise, with documented successful outcomes. Here, we present a case of EIA with lipid transfer protein (LTP) sensitization successfully treated with omalizumab with long-term follow-up. A 12-year-old girl presented to our allergy department because of recurrent episodes of EIA, with no specific food ingestion before exercise. Allergen testing revealed sensitization to weed pollens, particularly mugwort (76.1 kUA/L) and *Alternaria alternata* (10.8 kUA/L). Allergen component testing indicated sensitization to LTP components from mugwort Art v 3 (49.9 kUA/L), wheat Tri a 14 (2.03 kUA/L), and peach Pru p 3 (11.5 kUA/L), with a negative result for omega-5 gliadin. Despite initial prophylactic treatment with budesonide–formoterol (80/4.5 μg) and cetirizine (10 mg) before exercise, the patient still experienced EIA; she was then recommended for dupilumab therapy (an initial dose of 600 mg, followed by 300 mg every 2 weeks for six doses). However, even while undergoing dupilumab therapy, she suffered two anaphylactic episodes after running 800–1,000 m. With the patient's consent, a trial of omalizumab was initiated (injections of 300 mg every 4 weeks). After 2 months of omalizumab therapy, the patient showed significant improvement. She had been engaging in physical exercise three times a week and experienced a mild episode of urticaria. There were no further episodes of anaphylaxis or emergency room visits. By the fourth month of omalizumab treatment, she was able to consume food normally even just before exercising and had returned to her full activity level without any restrictions. This case presents the first successful off-label use of omalizumab in the prevention of EIA in the Chinese population. It is concluded that omalizumab may be helpful in resolving EIA symptoms, as evidenced by this case of successful long-term use.

## Introduction

Exercise-induced anaphylaxis (EIA) is a rare and potentially life-threatening disorder. The syndrome is often categorized into two groups: anaphylaxis caused exclusively by exercise and anaphylaxis caused by exercise after consuming a particular food (1). The latter entity is named food-dependent exercise-induced anaphylaxis (FDEIA) (2). Common foods implicated in FDEIA include wheat, shellfish, legumes, tree nuts, and tomatoes; other grains (such as maize and buckwheat), vegetables, and garlic are less commonly involved in FDEIA (1). For allergen components, our previous study identified omega-5-gliadin (Tri a 19) as the main allergen involved in Chinese wheat-dependent exercise-induced anaphylaxis (WDEIA) (3).

Lipid transfer protein (LTP) is responsible for the largest number of FDEIA cases in Mediterranean European countries but is rarely reported in China (4, 5). Omalizumab, an immunoglobulin E (IgE) monoclonal antibody well documented in allergic asthma and chronic spontaneous urticaria, has shown promise in small clinical trials for treating atopic dermatitis, food allergy, mast cell disorders, idiopathic anaphylaxis, and other allergic disorders (6). Dupilumab targets the alpha subunit common to both the IL-4 and IL-13 receptors (7). It is currently approved for moderate to severe asthma, atopic dermatitis, eosinophilic esophagitis, prurigo nodularis, and rhino sinusitis with nasal polyposis, and its use for additional indications is currently being investigated (7). In difficult to control and refractory cases of EIA, biologics such as omalizumab and dupilumab might be tried, and successful cases have been reported (8–12). In this report, we present a case of EIA with LTP sensitization successfully treated with omalizumab with long-term follow-up.

## Case report

A 12-year-old girl presented to our department after one severe episode of anaphylaxis. The severe episode occurred after a 1 h run. One hour before the initiation of the running she had eaten an apple and wheat bread. Her symptoms included itching, redness, generalized urticaria, facial angioedema, wheezing, and hypotension. She was successfully treated with intramuscular epinephrine (0.3 mg) and thereafter with intravenous methylprednisolone and antihistamine together with inhalation of a short-acting anticholinergic and β2-sympathomimetic agonist. Laboratory tests, including complete blood count, C-reactive protein, renal and liver function, were normal. Her medical history included allergic rhinitis since the age of 9 years old, recurrent chest distress, shortness of breath, and wheezing during exercise. The patient's mother had a history of allergic rhinitis. She was referred to the allergy department, where total IgE and specific IgE tests were conducted using ImmunoCAP (Thermo Fisher Scientific, Sweden). Results indicated elevated total IgE levels (506 kU/L) and sensitization to weed pollens, particularly mugwort (76.1 kUA/L), and *Alternaria alternata* (10.8 kUA/L). In addition, positive specific IgE was observed for common foods such as soybean, egg, milk, wheat, and peanut. Furthermore, LTP components from mugwort Art v 3 (49.9 kUA/L), wheat Tri a 14 (2.03 kUA/L), and peach Pru p 3 (11.5 kUA/L) were identified; testing for omega-5 gliadin was negative (Table 1). Pulmonary function tests were normal but the fractional exhaled nitric oxide (FENO) level was elevated (48 ppb). Skin prick tests were positive for apple (grade 2+) and maize flour (grade 4+) and negative for mango, pineapple, pear, strawberry, litchi, orange, peach, watermelon, pitaya, melon, cherry, rice, and wheat.

**Table 1:** *In vitro* allergy diagnostics: specific IgE antibody detection results (ImmunoCAP).

	19 October 2020 (first visit)	27 August 2021 (follow-up 10 months)	8 March 2022 (follow-up 1 year 5 months)	4 August 2022 (follow-up 1 year 10 months)	9 March 2023 (follow-up 2 years 5 months)
Allergen and allergen component	IgE (kUa/L)	IgE (kUa/L)	IgE (kUa/L)	IgE (kUa/L)	IgE (kUa/L)
T-IgE	506	333	206	1,338	1,112
Aeroallergens					
d1 (*Dermatophagoides pteronyssinus*)	0.06 (Neg)	0.2 (Neg)	0.1 (Neg)	0.8	0.85
d2 (*Dermatophagoides farina*)	0.07 (Neg)	0.16 (Neg)	0.06 (Neg)	0.76	0.78
t6 (mountain juniper)	ND	ND	3.37	14.6	10.1
w22 (Japanese hop)	ND	ND	16.4	81.7	85.8
w6 (mugwort)	76.1	25.6	16.6	74.8	68.7
m6 (*Alternaria alternata*)	10.8	10.7	5.07	38.2	33.8
t3 (common sliver birch)	0.52	0.4	0.15 (Neg)	0.52	0.31 (Neg)
t11 (maple leaf sycamore)	ND	ND	0.78	1.68	1.31
w1 (common ragweed)	1.37	0.32	0.28 (Neg)	1.13	1.33
g2 (Bermuda grass)	ND	ND	0.09 (Neg)	0.27 (Neg)	0.15 (Neg)
g6 (Timothy grass)	ND	ND	0.02 (Neg)	0.08 (Neg)	0.07 (Neg)
Foods					
f14 (soybean)	0.59	0.15 (Neg)	0.13 (Neg)	0.34 (Neg)	ND
f1 (egg white)	0.42	0.28 (Neg)	0.09 (Neg)	0.07 (Neg)	ND
f2 (milk)	0.62	0.46	0.15 (Neg)	0.20 (Neg)	ND
f4 (wheat)	0.62	0.25 (Neg)	0.17 (Neg)	0.48	ND
f13 (peanut)	8.17	1.49	1.94	5.57	ND
f23 (crab)	0.02 (Neg)	0.09 (Neg)	0.02 (Neg)	0.31 (Neg)	ND
f24 (shrimp)	0.06 (Neg)	0.09 (Neg)	0.05 (Neg)	0.39	ND
Allergen components					
f420 (rPru p3, LTP, peach)	11.5	ND	ND	ND	ND
f416 (rTri a19, omega-5 gliadin, wheat)	0	ND	ND	ND	ND
f433 (rTri a14, LTP wheat)	2.03	ND	ND	ND	ND
w231 (nArt v1 mugwort)	0.38	ND	ND	ND	ND
w233 (nArt v3 mugwort)	49.9	ND	ND	ND	ND

ND, not done; Neg, negative.

**Figure 1:**
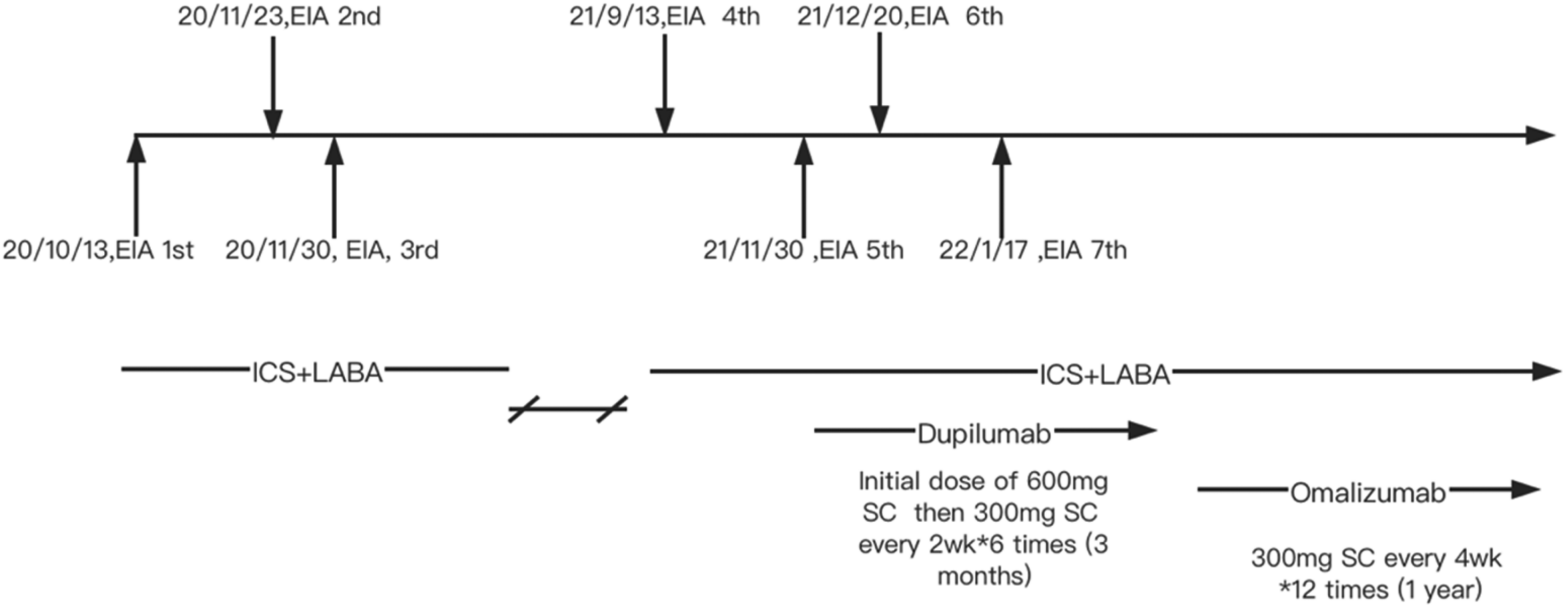
Clinical management of the patient. EIA, exercise-induced anaphylaxis; ICS + LABA, inhaled corticosteroids + long acting β agonists; SC, subcutaneous injection.

Although pulmonary function was normal, because of the recurrent wheezing during exercise and the elevated FENO level, the patient was initially prescribed budesonide–formoterol (80/4.5 μg) twice daily and advised to avoid consuming apple and maize before running. In addition, she was instructed to stop exercising immediately if she experienced flushing, itching, and hives. Despite following these recommendations, she suffered a second episode of anaphylaxis 8 weeks later (November 2020) after running for 30 min. She had eaten a full meal 1 h before exercise but could not recall what foods she had consumed. Her symptoms included generalized hives, itching, and shortness of breath. She was taken to the emergency room, where she received oral steroids (30 mg prednisone). Following this incident, she was prescribed budesonide–formoterol (80/4.5 μg) and cetirizine (10 mg), to be taken before exercise. In addition, she was advised to refrain from eating all foods 4 h before physical activity. Diet and activity were very difficult for this young junior high school student. Ten months later (September 2021), she continued to have breakthrough anaphylaxis reactions after routine running in gym class. In November 2021, she experienced an episode characterized by severe generalized hives during gym class. Before this episode, she had eaten several foods for lunch but could not recall the specific items she consumed. Given her refractory symptoms, she was referred to the dermatology department. The underlying Th2 inflammation mechanisms of EIA suggest that dupilumab therapy could be beneficial for this patient. With the consent of the patient and her parents, she was advised to initiate dupilumab therapy. The initial dose was 600 mg, followed by 300 mg every 2 weeks for a total of six doses from November 2021 to February 2022. However, even while undergoing dupilumab therapy, she suffered two anaphylactic episodes after running 800–1,000 m. The first episode occurred approximately 4 weeks into treatment, and the second episode occurred approximately 8 weeks into treatment (Figure 1). Owing to the worsening symptoms, despite preventive measures, the lack of response to dupilumab therapy, and the patient’s desire to resume normal activities, her mother wanted to try an alternative treatment. There were reports in the literature suggesting the effectiveness of omalizumab in treating EIA; however, this was off-label use. With the patient's consent, a trial of omalizumab was initiated. The patient was initiated on omalizumab injections of 300 mg every 4 weeks starting in March 2022 (Figure 1). The dosage was determined based on the patient's weight and total IgE levels, in accordance with the protocol used for asthma treatment. During the initial month of omalizumab therapy, the patient was advised to discontinue running. After 2 months of omalizumab therapy, the patient reported a significant improvement. She began engaging in physical exercise three times a week and experienced a mild episode of urticaria. There were no episodes of anaphylaxis or visits to the emergency room. By the fourth month of omalizumab treatment, she was able to consume food normally even just before exercising and had returned to her full activity level without any restrictions. Throughout the follow-up period, we monitored the total IgE and specific IgE levels, as shown in Table 1 and Supplementary Figure S1. The FENO level and variables of pulmonary function testing were also monitored during the follow-up period (Supplementary Figure S2).

## Discussion

EIAn and FDEIAn are clinical diagnoses, in this patient, anaphylactic symptoms occur during exercise, despite the patient showing multi-sensitization to several food allergens and LTP, which has been demonstrated as the main elicitor of FDEIA and food-dependent NSAID-induced hypersensitivity (FDNIH) in the Mediterranean region (5), anaphylaxis can still occur without specific food ingestion in this patient, resulting in a diagnosis of EIA. In this case, an exercise challenge was not conducted due to the inability to obtain informed consent from the child and her parents.

The pathophysiology of EIA and FDEIA is not fully understood. In addition to IgE-mediated mast cell degranulation, other proposed mechanisms include exercise-induced changes in plasma osmolality, alterations in blood pH, alterations in tissue transglutaminase, a redistribution of blood flow and antigen presentation, and changes in gut permeability (1). Current preventative treatments include exercise avoidance, food avoidance in FDEIA, and pretreatment with medications such as antihistamines, cromolyn, or montelukast before exercise (1). However, the pretreatment regimen did not prevent anaphylactic episodes in difficult to control and refractory EIA and FDEIA cases. In recent years, biologic agents (e.g., omalizumab and dupilumab) have been widely used. Although biologic agents are still not licensed for the management of anaphylaxis, these drugs, particularly omalizumab, appear to be effective as a preventive measure for EIA. We summarized the published data in Table 2. The suspected potential underlying mechanisms include omalizumab binding to free IgE, blocking the binding of IgE to its specific high-affinity receptor, and its mast cell stabilizing effect likely contributing to the clinical response. Recently, Zhu et al. (13) reported an 11-year-old Chinese boy who suffered recurrent EIA with mushroom intake. After seven subcutaneous injections of dupilumab, the patient was exposed to the culprit mushrooms and exercised at least twice a month without notable anaphylaxis, suggesting that dupilumab may improve allergic reactions in FDEIA patients by blocking the IL-4Rα signaling pathway.

**Table 2:** The use of biologics in EIA and FDEIA management (cases published in English literature).

Author (years)	No.	Age (years)	Sex	Atopic conditions	Diagnosis	Prophylactic regimen before biologics	Biologics	Dose and frequency	Frequency of anaphylaxis before biologics	Frequency of anaphylaxis after biologics
Christensen and Bindslev-Jensen (8)	1	17	M	None	EIA (challenge confirmed)	Epi prn	Omalizumab	300 mg every 2 weeks × 4 months, and then increase the dose interval	Recurrent episodes of generalized urticaria, 1 anaphylaxis episode	0
Bray et al. (9)	1	14	M	AR	EIA	Montelukast; epi prn; fexofenadine, cromolyn, and ranitidine 2 h before exercise	Omalizumab	300 mg monthly × 4 months	2 anaphylaxis episodes	0
Peterson and Coop (10)	1	39	M	Eosinophilic esophagitis, AR	EIA	Fexofenadine, montelukast, and ranitidine, epi prn	Omalizumab	300 mg every 2 weeks × 3 months	Frequent episodes	0
Mobayed et al. (11)	1	38	F	None	EIA	Levocetirizine–montelukast	Omalizumab	300 mg monthly × 4 months	Frequent episodes (10–12 per month in a hot and humid climate	0
Zhu et al. (13)	1	11	M	AS, AR, AC	FDEIA	None	Dupilumab	300 mg every 4 weeks × 3 times then changed to every 6–7 weeks × 4 times	10	2 times of mild reactions

M, male; F, female; EIA, exercise-induced anaphylaxis; FDEIA, food-dependent exercise-induced anaphylaxis; AR, allergic rhinitis; AS, asthma; AC, allergic conjunctivitis.

The geographical distribution of pollen allergens and regional dietary habits influence plant-derived food trigger sensitization profiles and clinical symptoms. In Northern China, mugwort and Japanese Hop pollen are common pollens causing seasonal respiratory allergies in autumn. Despite the patient's multiple sensitizations to wheat, soy, and peanut, there were no allergic reactions upon consuming these foods. This could be attributed to pollen-food cross-reactivity or was potentially a false positive due to high total IgE.

Based on published cases and our findings, it appears that omalizumab may be a more effective choice as a biologic than dupilumab when prophylactic measures fail to achieve good control of EIA. However, it is important to note that our study is limited to a single case, which highlights the need for further research. One major limitation of our case study was that the diagnosis of EIA was based on clinical manifestations rather than a challenge test. In addition, owing to limitations in the detection reagent for tryptase in our laboratory, we were unable to measure the level of tryptase. Furthermore, a longer follow-up period is necessary to determine whether the effectiveness of the treatment persists.

This report presents the first successful off-label use of omalizumab in the prevention of EIA in the Chinese population. It is concluded that omalizumab may be helpful in resolving EIA symptoms, as evidenced by this case of successful long-term use. We suggest that mast cell stabilization may be the main contributing factor in reducing the symptoms of EIA, but more research needs to be carried out to validate this presumption. In addition, we recommend further clinical studies of the treatment of EIA with omalizumab.

## Data Availability

The original contributions presented in the study are included in the article/Supplementary Material, further inquiries can be directed to the corresponding author.

## Supplementary material

Supplementary Figure 1 The level of total IgE and specific IgE for common aeroallergen and food allergens during follow up period.

Supplementary Figure 2 The level of fractional exhaled nitric oxide (FENO) and variables of pulmonary function testing during follow up period.
