# Supplementary figures and images for: The management of exercise-induced anaphylaxis in a Chinese child with biologics: a case report
Source: Front Allergy. 2024 Sep 19;5:1453873. doi: 10.3389/falgy.2024.1453873 (PMC11446895; doi:10.3389/falgy.2024.1453873)

## Slide 1
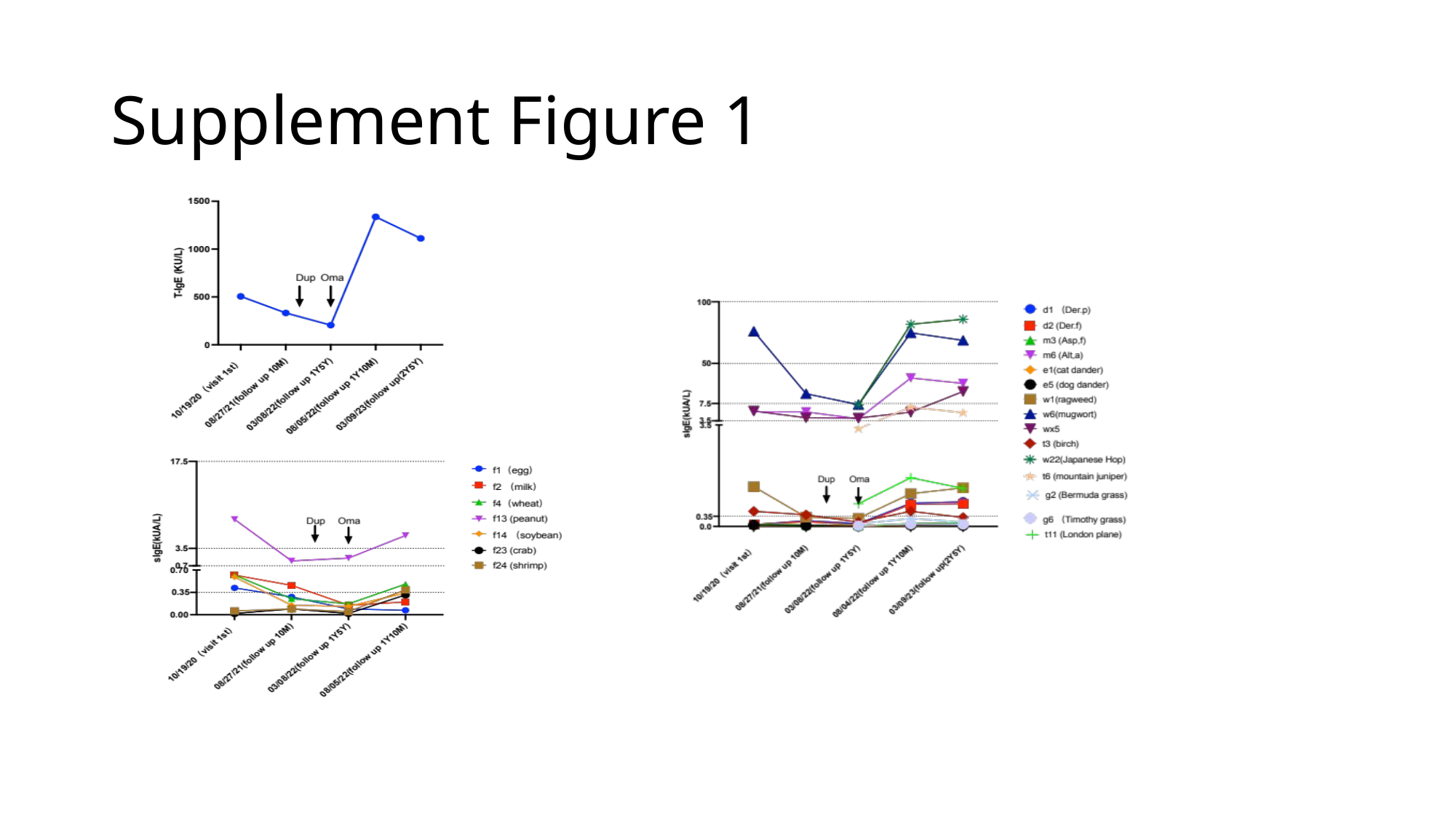

# Supplement Figure 1

Supplement: Supplementary Figure 1 — The level of total IgE and specific IgE for common aeroallergen and food allergens during follow up period. [file Presentation1.pptx]

## Slide 1
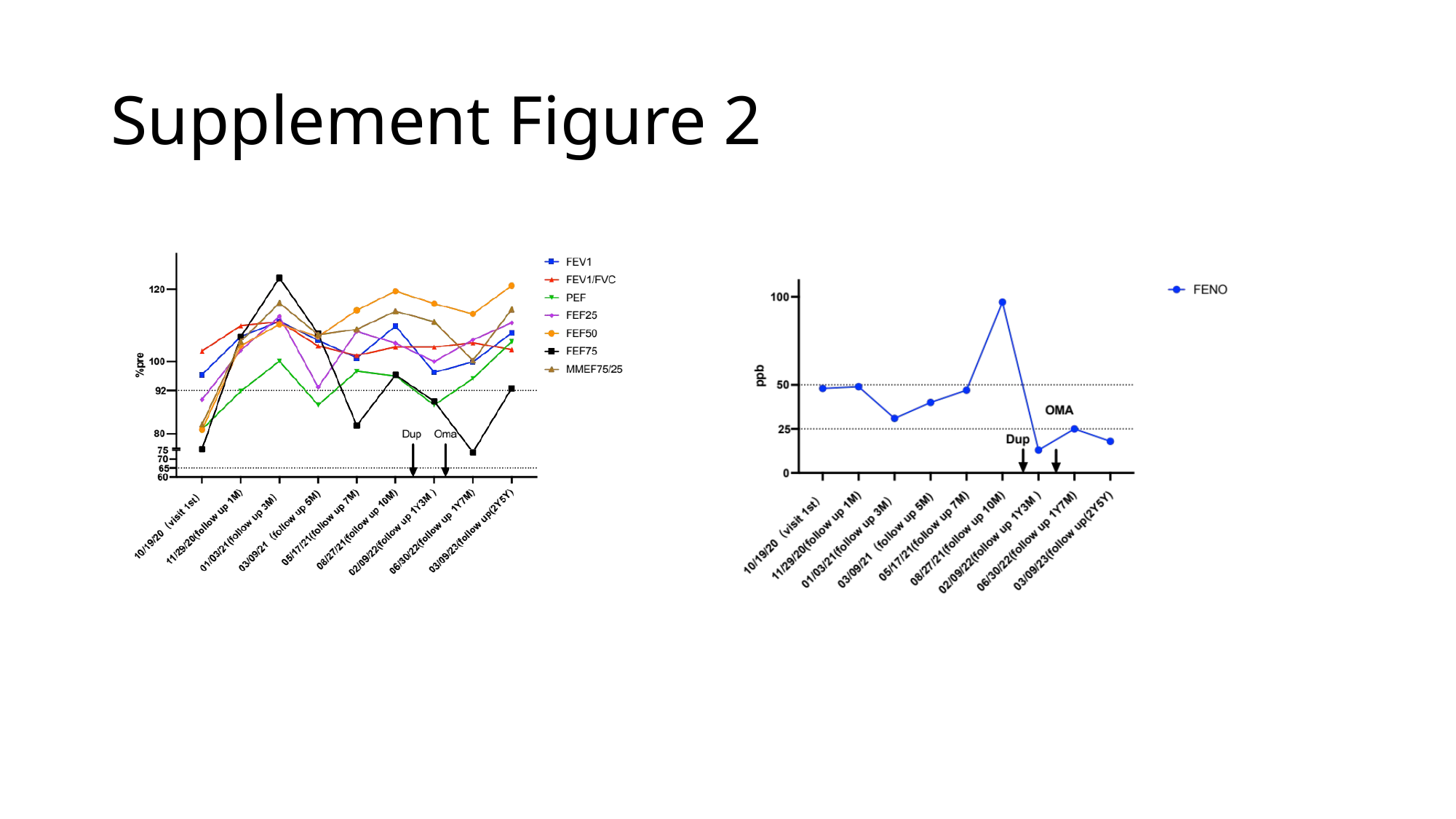

# Supplement Figure 2

Supplement: Supplementary Figure 2 — The level of fractional exhaled nitric oxide (FENO) and variables of pulmonary function testing during follow up period. [file Presentation2.pptx]
